# Case Report: Interferon-Alpha-Induced Neuromyelitis Optica Spectrum Disorder

**DOI:** 10.3389/fneur.2022.872684

**Published:** 2022-04-25

**Authors:** Jie Rao, Na Xu, Jing Sun, Yan Li, Fangwang Fu

**Affiliations:** ^1^Department of Neurology, The Second Affiliated Hospital and Yuying Children's Hospital of Wenzhou Medical University, Wenzhou, China; ^2^Department of Neurology, The Fifth Affiliated Hospital of Wenzhou Medical University, Lishui, China

**Keywords:** NMOSD, AQP4 antibody, interferon, drug-induced disease, autoimmune disease, case report

## Abstract

**Background and Objectives:**

To describe a new case of neuromyelitis optica spectrum disorder (NMOSD) induced by the administration of interferon-alpha (IFNα) and to raise awareness of this rare drug-induced disease of IFNα treatment.

**Methods:**

A single case study and comprehensive literature review of eight cases.

**Results:**

A 24-year-old man was diagnosed with cerebral venous thrombosis and essential thrombocythemia. He had been undergoing IFNα treatment (IFNα-2b, 3 million IU per day) without any side effects for 18 months, at which point the patient developed persistent hiccups, nausea, urinary retention, and numbness. Spinal magnetic resonance imaging revealed a longitudinal abnormality extending from the medulla to the entire spinal cord. The patient was positive for anti-aquaporin-4 antibody (AQP4-IgG) in both the serum and cerebrospinal fluid (CSF), which confirmed the diagnosis of NMOSD. Thus, recombinant IFNα-2b was suspended immediately. Because his condition did not improve after 6-day treatment of methylprednisolone pulse therapy (1,000 mg for 3 days, then 500 mg for 3 days), intravenous immunoglobulin (0.4 g/kg/day for 5 days) was administered. The patient gradually improved. Low-dose prednisolone and mycophenolate mofetil were subsequently administered as a long-term treatment. The patient was discharged with subtle limb numbness and their expanded disability status score (EDSS) was 1. At the 1-year follow-up, the patient had not relapsed and tested negative for AQP4-IgG. We further identified the eight patients with IFNα-induced NMOSD. The median onset age was 59 years, and the median time of IFNα exposure was 18 months. Optic neuritis was the most common initial symptom (five, 55.6%), followed by myelitis in three patients and area postrema syndrome in one patient. More than half (five, 55.6%) of the patients were monophasic. After IFNα discontinuation and immunotherapy, most (seven, 77.8%) patients remained relapse-free. However, only one patient was free of sequelae.

**Conclusion:**

This study highlights the potential pathogenic risk of NMOSD of IFNα treatment. Given the high disability rates of this rare drug-induced disease, it is crucial to monitor the early manifestations of NMOSD during IFNα treatment.

## Introduction

Neuromyelitis Optica spectrum disorder (NMOSD) refers to a spectrum of the central nervous system (CNS) neuroinflammatory demyelinating disease that predominantly attacks the spinal cord, optic nerves, and brain (1, 2). The NMOSD is usually associated with a significant reduction in patients' quality of life and heavy economic burden. In contrast to T-lymphocyte-predominant myelinopathy in multiple sclerosis (MS) (3), NMOSD is considered an autoimmune astrocytopathy mediated by anti-aquaporin-4 antibodies (AQP4-IgG) (2). Type I interferons (IFNIs), particularly interferon-alpha (IFNα), are widely used to treat various infectious, immunological, and oncological diseases (4). They are also moderately effective for restricting CNS autoimmunity and are currently recommended as first-line immunomodulatory therapy for MS (5, 6). However, their use in patients with NMOSD is associated with neurologic deterioration and increased AQP4-IgG production (7, 8), which suggests a possible link between the development of NMOSD and IFNI. Although various CNS side effects of IFNI treatment have been reported to date, NMOSD remains largely unrecognized as a drug-induced disease (DID) of IFNI therapy (9). Recently, NMOSD cases associated with IFNα treatment started arising sporadically in patients with hepatitis C (9–14), systemic mastocytosis (9), and malignant melanoma (15). However, the underlying diseases may contribute to the occurrence of NMOSD in such cases. The precise relationship between IFNα and NMOSD remains elusive.

Herein, we present a case where the patient developed NMOSD following the administration of recombinant IFNα-2b for essential thrombocythemia (ET). We also determined the clinical profile and potential mechanism of IFNα-induced NMOSD by reviewing previously published cases.

## Methods

This study was a single case report with a comprehensive literature review. The ethics committees waived institutional review and ethical approval. The patient provided written informed consent for publication. De-identified data are available upon appropriate request to the corresponding author.

We performed a systematic review of reported cases published between January 1, 2000, and December 31, 2021, by searching PubMed and China National Knowledge Infrastructure databases using the following search strategy: “NMOSD,” “NMO,” “AQP-4 antibody,” “type I interferon,” “interferon-alpha,” and “interferon-beta.” Reference lists of the identified publications were manually searched to find relevant publications not captured by the initial search strategy. Seven reports of eight cases were identified and reviewed.

## Case Presentation

**Figure 1:**
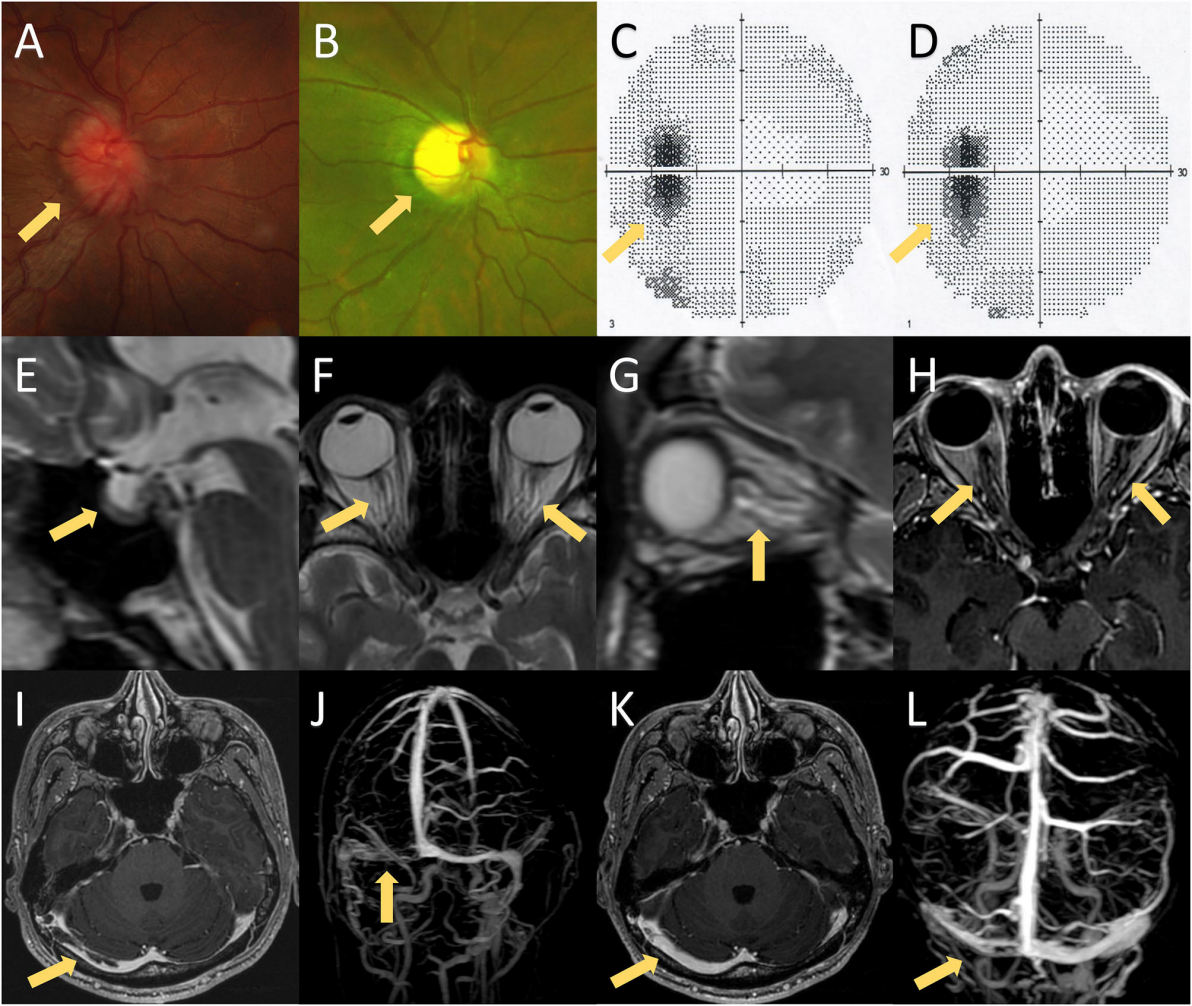
MRI findings, fundoscopic findings, and visual fields of the presented case. **(A)** Fundoscopic examination on the first hospitalization showed bilateral papilledema. **(B)** Fundoscopic examination on the second hospitalization revealed bilateral optic atrophy, which was probably caused by bilateral papilledema. **(C,D)** Visual fields on the first and second hospitalizations showed bilateral enlargement of the physiological blind spot without any other visual field defect. **(E–G)** Brain MRI exhibited several imaging signs of intracranial hypertension, including the empty-sella sign **(E)**, perioptic subarachnoid space distension **(F,G)**, optic nerve tortuosity **(G)**, and posterior globe flattening **(F,G)**. **(H)** Brain MRI with contrast showed no enhancement of the optic nerve. **(I,J)** Post-contrast 3D GRE T1-weighted imaging and contrast-enhanced magnetic resonance venography (CE-MRV) showed filling defects of the right transverse-sigmoid sinus. **(K,L)** Brain MRI and CE-MRV after 3 months of anticoagulation showed partial recanalization of the right transverse-sigmoid sinus.

**Figure 2:**
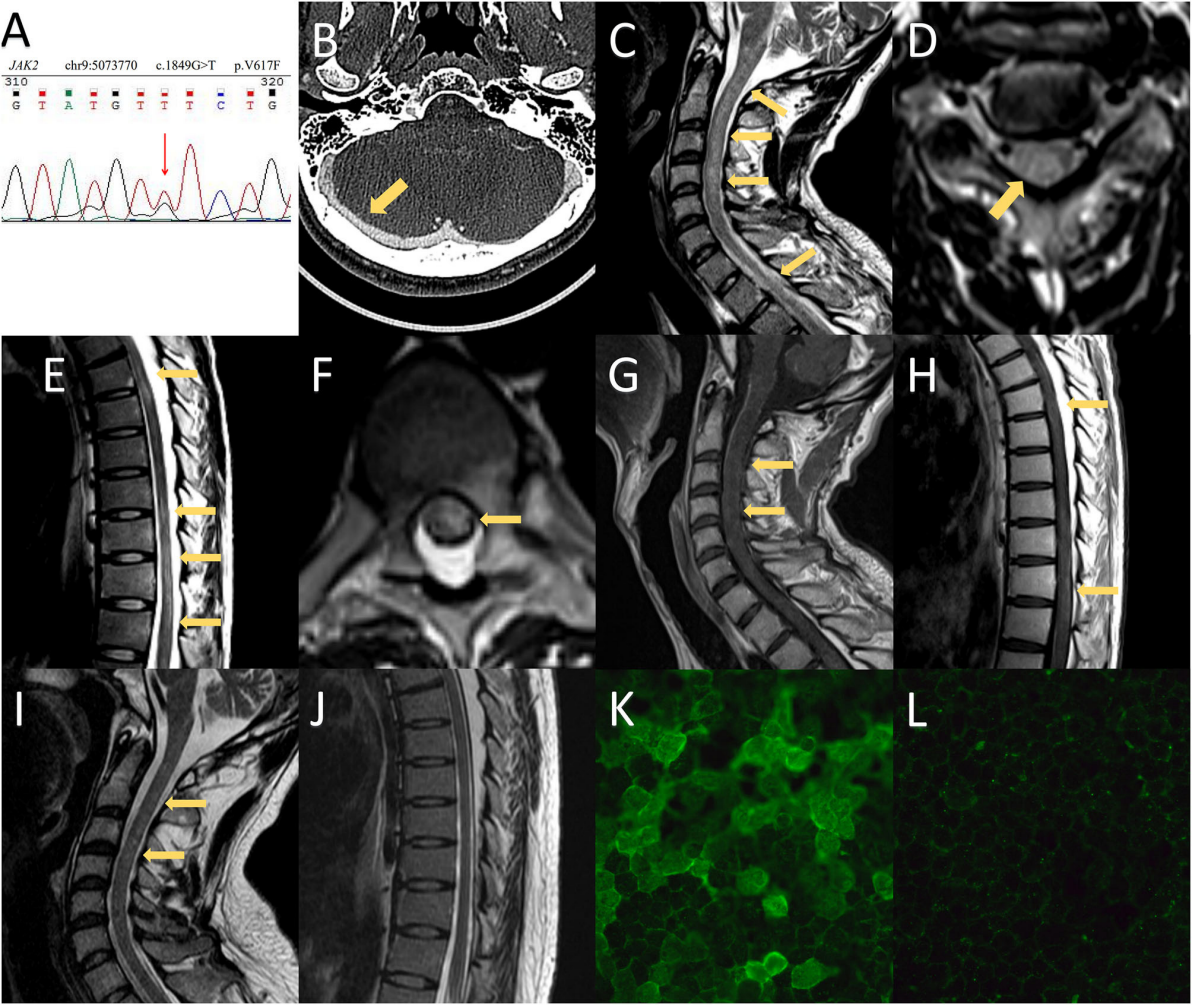
MRI and CT findings and anti-aquaporin-4 antibody (AQP4-IgG) array of the presented case. **(A)** Whole-exome sequencing revealed a mutation in JAK2-V617F. **(B)** CT venography of the second hospitalization showed no filling defects of the cerebral venous sinus. **(C–H)** Spinal MRI demonstrated T2-weighted imaging abnormalities in the medulla as well as extensive spinal cord involvement extending from C1 to the conus. Spinal MRI with contrast showed mild enhancement in the cervical and thoracic spinal cord. **(I,J)** Follow-up MRI showed prominent regression of the hyperintense lesion, which included the medulla and spinal cord. **(K)** The serum titer of AQP4-IgG using a cell-based assay was 1:1,000 at the time of neuromyelitis optica spectrum disorder diagnosis. **(L)** The patient was negative for AQP4-IgG at the 1-year follow-up.

A 24-year-old Chinese man presented with progressive headaches and right tinnitus for 3 months. He had no medical or family history relevant to his symptoms. He first visited the local hospital 2 weeks after onset, but no neurologic deficit was found. Brain MRI was performed 1 month later, which showed no abnormalities. He was admitted to our hospital after developing nausea and blurred vision. Neurological examination showed neck stiffness and bilateral optic disc swelling (ODS) (Figure 1A). Bilateral enlargement of the physiological blind spot was found in the visual field examinations (Figure 1C). Brain MRI with contrast showed no enhancement of the optic nerve (Figure 1H); however, the patient exhibited several imaging signs of intracranial hypertension (IH), which included the empty-sella sign (Figure 1E), perioptic subarachnoid space distension (Figures 1F,G), optic nerve tortuosity (Figure 1G), and posterior globe flattening (Figures 1F,G). A lumbar puncture was subsequently performed with an opening pressure of over 400 mm H_2_O. The CSF analysis results were all within normal ranges. Therefore, the ODS was considered a result of IH rather than optic neuritis. Contrast MRI also showed filling defects in the right transverse-sigmoid sinus (Figure 1I), which were confirmed by contrast-enhanced magnetic resonance venography (CE-MRV) (Figure 1J). The diagnosis of cerebral venous sinus thrombosis (CVST) was then established. Thrombosis risk factors were screened, and laboratory analyses for acquired and genetic hypercoagulability states were performed. However, the patient showed no abnormalities except for thrombocytosis (402 × 10^9^ /L). The patient underwent whole-exome sequencing, which revealed a mutation in JAK2-V617F (Figure 2A). Furthermore, a bone marrow biopsy showed increased numbers of megakaryocytes. Thus, ET with high thrombosis risk was established. The patient received low-molecular-weight heparin for 1 month, then, switched to indefinite 3-month warfarin treatment with an INR of 2–3. For patients with ET in the high-risk category, timely initiation of first-line cytoreduction therapies with recombinant IFNα or hydroxyurea are recommended. Therefore, IFNα-2b (3 million IU per day) was prescribed, and platelet count was controlled adequately. Headache and blurred vision gradually subsided within 2 months. Two months later, a repeat MRI showed partial recanalization of the right transverse-sigmoid sinus (Figures 1K,L), and the CSF opening pressure was 200 mm H_2_O. Repeated fundoscopic examination showed mild improvement of ODS; thus, acetazolamide (250 mg twice a day) was administered to control IH.

The patient had no side effects of IFNα treatment, and his condition remained stable. However, 18 months later, he complained of persistent hiccups, nausea, and vomiting for 8 days and was admitted to the gastroenterology department on the 9th day for gastrointestinal endoscopy and abdominopelvic CT. However, no abnormalities were observed. The patient presented with acute urinary retention on day 12. Neurological consultation was requested following the lack of relief from antiemetic therapy. Given the high clinical suspicion of CVST recurrence, MRI, CT venography, lumbar puncture, and fundoscopic examinations were suggested. Fundoscopic and visual field examination on the 12th day showed bilateral optic atrophy and enlarged blind spot, which were considered a result of prolonged IH rather than acute IH (Figures 1B,D). However, the lack of filling defects of the cerebral venous system in CT venography ruled out the recurrence of CVST on the 13th day (Figure 2B). The patient developed numbness of the extremities and tingling around both calves on day 14 and was transferred to the neurology department. Neurological examination showed that he was conscious and oriented with horizontal nystagmus toward the left side. He had grade 5/5 strength and normal muscle tone; however, tendon hyperreflexia was observed in all four extremities. Sensory examination showed impaired proprioception sensations in both legs. Babinski and Hoffman's sign was positive bilaterally. Signs of meningeal irritation were absent. Repeated lumbar puncture was performed on the 15th day with a CSF opening pressure of 210 mm H_2_O. The CSF examination showed only mild mononuclear pleocytosis (80/ul) without abnormalities in proteins, immunoglobulin G (IgG) index, or oligoclonal bands. Spinal and brain MRI with contrast was undertaken on the 17th day, which showed an extensive signal abnormality that extended longitudinally from the medulla to the entire spinal cord, with slight edema and gadolinium enhancement (Figures 2C–H).

Serology for rheumatoid factor, anti-neutrophil cytoplasmic antibodies (ANCA), antiphospholipid antibodies, anti-Sjögren syndrome A/B antibodies, paraneoplastic panel, human leukocyte antigen (HLA) B5, and HLA-B51 were all negative. Serum and CSF infection panels were also negative. Antinuclear antibody (ANA) was positive at a titer of 1:100. Cytokines panel showed elevated interleukin-6 (IL-6) at 19 pg/ml (normal range 0–3 pg/ml). The cell-based AQP4-IgG assay of serum and CSF were positive at 1:1,000 and 1:100 (Figure 2K), respectively. Moreover, myelin-oligodendrocyte-glycoprotein IgG and glial-fibrillary-acidic-protein IgG were negative. Thus, the diagnosis of NMOSD was established according to the 2015 NMOSD criteria, and IFNα-2b treatment was suspended immediately. On the 18th day, the patient experienced weakness of both legs with an Expanded Disability Status Score (EDSS) of 3. However, neurological examination demonstrated only mild distal leg weakness (plantar flexion and ankle dorsiflexion, grade 4/5 strength). We administered a 12-day treatment plan with methylprednisolone (1,000 mg for 3 days, 500 mg for 3 days, 240 mg for 3 days, and 120 mg for 3 days). However, his clinical symptoms did not improve after 6 days of treatment. Intravenous immunoglobulin (0.4 g/kg/day for 5 days) was administered on the 23rd day, followed by mycophenolate mofetil (0.5 g twice a day) and oral prednisolone (60 mg per day, reduced to 20 mg per day step by step) as prophylaxis. In addition, hydroxyurea and aspirin were given instead of IFNα-2b to control ET.

The patient gradually improved, and urinary retention and lower limb weakness diminished on the 27th day. Hiccups and nausea subsided on the 31st day, and the patient was discharged on the 32nd day. The patient only had slight limb numbness at discharge, with an EDSS score of 1. On the follow-up visit 1 year later, the patient remained symptom-free and relapse-free, and his EDSS score was 0. Spinal MRI revealed that most foci had recovered (Figures 2I,J). Serum AQP4-IgG (Figure 2L) and ANA tests were negative. Long-term administration of mycophenolate mofetil and low dosage prednisolone was maintained. The patient remained symptom-, relapse-, and side-effect-free until December 2021.

## Review of the Published Literature and the Present Case

Our literature review identified six case reports and one small case series. Together with our patient, nine patients with IFNα-induced NMOSD, who met 2015 NMOSD criteria were included for the pooled analysis (1). The clinical summary is provided in Table 1. The median age of NMOSD occurrence in patients was 59 years (range: 24–65 years), and 55.6% (5/9) of patients were women. More than half of patients were characterized by relapsing NMOSD. Hepatitis C infection was the most common primary disease (six patients, 66.7%). The remaining two cases had malignant melanoma and systemic mastocytosis, respectively, whereas our patient had ET as the primary disease. Optic neuritis (ON) was the most common initial symptom (five patients, 55.6%), followed by myelitis in three patients and area postrema syndrome (APS) in one patient. Six patients presented with ON, six presented with transverse myelitis, and only one patient had APS. Four patients presented with brain lesions in MRI, of whom three were asymptomatic. Two patients were noted as having slight pleocytosis in the CSF. Correspondingly, these patients also had a slight-to-moderate increase in proteins in the CSF. Oligoclonal bands were not found in any patient. Serum AQP4-IgG was positively detected in all patients.

**Table 1:** Characteristics of the patients with Interferon-alpha-induced neuromyelitis optica spectrum disorder (NMOSD).

**Study (references)**	**Age/sex**	**Primary** **disease**	**IFNI** **type**	**IFNI** **exposure**	**Initial** **symptom**	**Clinical** **phenotype**	**MRI** **findings**	**CSF**	**Other** **antibody**	**Acute** **treatment**	**Long-term** **treatment**	**Relapse**	**EDSS**	**Outcome**
Kajiyama et al. (14)	47/F	CHC	IFNα-2b	13 M	ON	ON+TM	Brain:periventricular WM Spinal: 4th thoracic TM	N/A	Negative	CS, CR	CS	No	N/A	N/A
Yamasaki et al. (11)	65/F	CHC	IFNα-2b IFNα-2a	34 M	ON	ON+ACS	Brain: callosum, WM, → cerebral pyramidal tract lesion Spinal: (–)	Negative	Negative	IVMP, CR	CS	Yes, 3 times	1	Visual defect
Kawazoe et al. (10)	60/F	CHC	IFNα IFNα-2b IFNα-1 IFNβ IFNα-2a	180 M	ON	ON	Brain: WM ON: left ON Spinal: (–)	Negative	Anti-GAD	1st: Oral CS, NE 2nd: IVMP, NE PE+IVMP, PR IVIG+CPM, PR	1st: MTX 2nd: monthly CPM	Yes, 2 times	1	Visual defect
Usmani et al. (12)	62/M	CHC	IFNβ-1a IFNα	7 M	TM	TM	Spinal: LETM from the medulla to upper thoracic Brain: (–)	Elevated protein (>500 mg/dL)	Negative	IVMP, NE IVIG, NE	CS	No	8	Lower extremities paralysis
Mangioni et al. (13)	32/M	CHC	IFNα-2a	3 M	ON	ON+TM	Spinal: LETM of the entire spinal cord and lower medulla Brain: (–)	Elevated protein; Pleocytosis (15/μL)	Negative	IVMP, NE PE+IVIG, PR	CS	No	6	Paraplegia, proprioceptive sensibility defect
Gao et al. (15)	40/M	MM	IFNα-2b	55 M	ON	ON	Spinal: (–) Brain: (–)	Negative	Negative	IV DXM, PR IVMP, NE	CS, rituximab	No	5	Visual defect
Williams et al. (9)	65/F	SM	IFNα	120 M	TM	ON+TM	Spinal: LETM thoracic	N/A	Negative	CS	Azathioprine, rituximab	Yes	N/A	N/A
	59/M	CHC	IFNα	12 M	TM	TM	Spinal: LETM thoracic	N/A	Negative	CS	Azathioprine	Yes	N/A	N/A
Present case	24/M	ET	IFNα-2b	18 M	APS	APS+TM	Spinal: LETM of entire spinal cord Brain: medulla	Pleocytosis (80/μl)	ANA	IVMP+IVIG, CR	CS, MMF	No	0	Symptom-free and relapse-free

*ACS, acute cerebral syndrome; ANA, antinuclear antibodies; APS, area postrema syndrome; CHC, chronic hepatitis C; CR, complete remission; CS, corticosteroids; CPM, cyclophosphamide; DXM, dexamethasone; EDSS, expanded disability status score; ET, essential thrombocythemia; IFN, interferon; IFNI, type-I interferon; IVIG, intravenous immunoglobulin; IVMP, intravenous methylprednisolone; LETM, longitudinally extensive transverse myelitis; MM, malignant melanoma; MMF, mycophenolate mofetil; MTX, methotrexate; NE, not effective; ON, optic neuritis; PR, partial remission; SM, systemic mastocytosis; WM, white matter*.

All patients received IFNα, and only two patients had transient exposure to IFNβ. The median time of IFNα exposure was 18 months (range 3–180 months). Notably, two patients developed NMOSD after discontinuation of IFNα (after 2 and 3 months, respectively). Autoimmune antibodies secondary to IFNα treatment were detected in two patients: ANA and an anti-glutamate decarboxylase (GAD) antibody. Less than half (4/9) of patients responded favorably to initial immunotherapy. Of the six patients who had available outcome data, only one patient showed no sequelae. After IFNα discontinuation and long-term immunotherapy, most (seven patients, 77.8%) patients remained relapse-free. Only two patients reported transverse myelitis relapse following immunotherapy.

## Discussion

### Considerations of the Diagnostic Process of the Presented Case

We presented the first NMOSD case with ET, which is one of the myeloproliferative neoplasms (MPNs). Is MPN a potential cause of NMOSD? Immune dysregulation is an increasingly reported characteristic of MPNs (16). An association has already been reported between MPNs and several autoimmune diseases, including rheumatoid arthritis and systemic lupus erythematosus (SLE) (16). Galimberti and colleagues found that 7.8% of patients with an MPN presented with an overt autoimmune disease (16). Moreover, the unusual concurrence of MS and MPN has been reported in a Roskilde MPN population. However, all five patients developed MPNs after MS. The authors concluded that chronic inflammation in MS patients may contribute to the development of MPNs (17). To date, there are no reports describing the occurrence of NMOSD in patients with MPNs. These results suggest that the presence of MPNs is not sufficient to cause NMOSD.

In addition, we presented a specific case of NMOSD, which developed following the administration of IFNα with a significant diagnostic delay. Thus, several considerations are warranted. The ODS was the most prominent presence in the early stages of our case. Thus, we question whether ODS is an early manifestation of NMOSD. The most common causes of ODS include papilledema (resulting from intracranial hypertension), inflammatory ON, and anterior ischemic optic neuropathy (18). The ODS of different etiologies usually appear ophthalmoscopically similar. Binocular involvement relatively preserved visual function, and symptoms, such as headache and tinnitus, can frequently help physicians distinguish papilledema from other diseases with ODS (18, 19). However, binocular ODS can emerge in ischemic and inflammatory optic neuropathies (18). Indeed, there are several case reports, in which bilateral ODS of NMOSD was misinterpreted as IH, even with marked elevated intracranial pressure. Furthermore, there are some cases, in which ODS with CVST was mistaken for ON. It can sometimes be challenging to determine the underlying cause of ODS (20); however, there are several helpful techniques. The optical coherence tomography (OCT) and OCT angiography (OCTA) provide non-invasive imaging of the optic circulation and can be used to identify ischemic ODS. The most sensitive diagnostic modality for ON is MRI with fat-saturated sequences and contrast enhancement (19). The abnormal enhancement of the optic nerve is direct evidence of ON. In addition, MRI of the orbits can reveal signs suggestive of IH, which include the empty-sella sign, perioptic subarachnoid space distension, optic nerve tortuosity, and posterior globe flattening (21). Our case presented with headache, tinnitus, ODS, preserved visual function, and a CSF pressure of more than 400 mm H_2_O, which strongly supported the diagnosis of papilledema. We also carefully re-analyzed the patient's neuroradiological data. Characteristic MRI findings for papilledema were identified without optic nerve enhancement. Therefore, in our case, bilateral ODS and secondary optic atrophyoptic atrophy were considered a consequence of CVST rather than the initial presentation of NMOSD.

In addition, our patient, who presented with APS as the initial symptom of NMOSD, was first admitted to the gastroenterology department. Intractable hiccups and vomiting are commonly encountered problems in the gastroenterology department or general medicine service (22). More than 70% of patients with APS are reported to visit a gastroenterologist or internist initially, and neurologic evaluation is not commonly pursued (22). However, intractable hiccups and vomiting can be a common clinical presentation of IH; although the absence of headache and papilledema made it an unlikely diagnosis. Thus, clinicians need to be aware of NMOSD as a diagnosable central cause for patients presenting with unexplained intractable nausea and vomiting.

### Is NMOSD a DID of IFNα Treatment?

The IFNα, a member of the IFNI family, plays a critical role in linking innate and adaptive immunity (23). The IFNIs exert their anti-proliferative, anti-tumor, anti-angiogenic, and immunomodulatory properties by activating the Janus kinase signal transducer and activator of transcription signals *via* the common type-I IFN receptor (IFNAR) (23, 24). The IFNα is still widely used in the treatment of chronic viral infections, hematological malignancies, and certain cancers (25), whereas IFNβ preparations are recommended for multiple isoforms of MS (5, 6). Opposing the beneficial actions of IFNI treatment, IFNI has gradually been recognized as a pro-inflammatory molecule that may not only unmask and aggravate underlying autoimmune processes, but also induce *de novo* autoimmune disorders, such as type-I diabetes, vitiligo, SLE, Sjögren syndrome, and autoimmune thyroid disease (26–28). Various neuroautoimmune diseases, including myasthenia gravis, inflammatory demyelinating polyneuropathy, and polymyositis, are also occasionally induced by IFNα therapy (29, 30).

Notwithstanding, NMOSD is still not formally regarded as a DID of treatment with IFNα. To date, few cases of IFNα-induced NMOSD have been reported, and this condition has likely been ignored by clinicians. Only eight cases with NMOSD secondary to IFNα therapy have been described to date. A few cases of IFNα-induced CNS demyelinating disease, including two cases with ON, two with ON and myelitis, and two with MS-like demyelinating disease, have been reported previously (15). However, AQP4-IgG was not detected in these cases, which hindered definite diagnoses. The first AQP4-IgG seropositive case of IFNα-induced NMOSD was reported in 2007 by Kajiyama et al. (14). The patient developed bilateral ON, transverse myelitis, and multiple periventricular white matter lesions after undergoing 13 months of IFNα treatment for chronic hepatitis C (14). Since then, nine patients have been reported with NMOSD with HCV infection as the primary disease, although three patients were not treated with IFNI (31). A possible association between NMOSD and HCV infection is likely; clinical data suggest that extrahepatic diseases are present in 40–74% of patients with hepatitis C as a result of complex interactions between HCV and B lymphocytes (31, 32). The HCV may contribute to immune system dysregulation, lymphocytes activation, and autoimmune antibody production, which include anti-ANA, anti-ANCA, and AQP4-IgG (32). Therefore, IFNα may form the bridge between NMOSD and HCV infection. Unfortunately, IFNα concentration was not measured in the NMOSD patients who did not undergo IFNα treatment.

In the remaining patients without HCV infection, IFNα was considered the independent trigger for the occurrence of NMOSD (9, 15). In addition, Williams and colleagues reported two cases in which NMOSD was secondary to exposure to significantly elevated endogenous IFNα, which was constitutively produced by the underlying interferonopathic disease, including genetic interferonopathy and SLE (9). The above evidence strongly supports the notion that NMOSD is a DID of IFNα treatment.

Interestingly, IFNβ treatment can exacerbate NMOSD but rarely induces newly onset NMOSD. In terms of mechanistic aspects, both IFNα and IFNβ may contribute to the development of NMOSD. The capacity to penetrate the blood-brain barrier (BBB) is an essential factor for DIDs of the CNS. A previous study showed that peripheral IFNα was able to exert its effects across the BBB (33). However, peripheral IFNβ was reported to have no direct access to an intact BBB (34).

The IFNα-induced NMOSD shares heterogeneous disease process patterns with AQP-IgG seropositive NMOSD (35), where clinical data, neuroradiological data, treatment response, and outcome features are largely similar across both groups. This is unsurprising because both conditions share the common pathogenetic AQP4-IgG pathway to NMOSD. Notably, the onset of NMOSD in some patients occurs several months after discontinuation of IFNα (12, 15). In an *in vitro* model, astrocytes showed markedly increased reactivity and dysregulation of the downstream gene and cytokines after exposure to IFNα for 3 weeks. Notably, these effects were not restored even 7 days after withdrawal of IFNα (36). Therefore, the prolonged exposure of IFNα may lead to persistent activation of the neuroautoimmune cascade even after the cessation of IFNα exposure.

#### The Role of IFNI in the Immunopathogenesis of NMOSD

**Figure 3:**
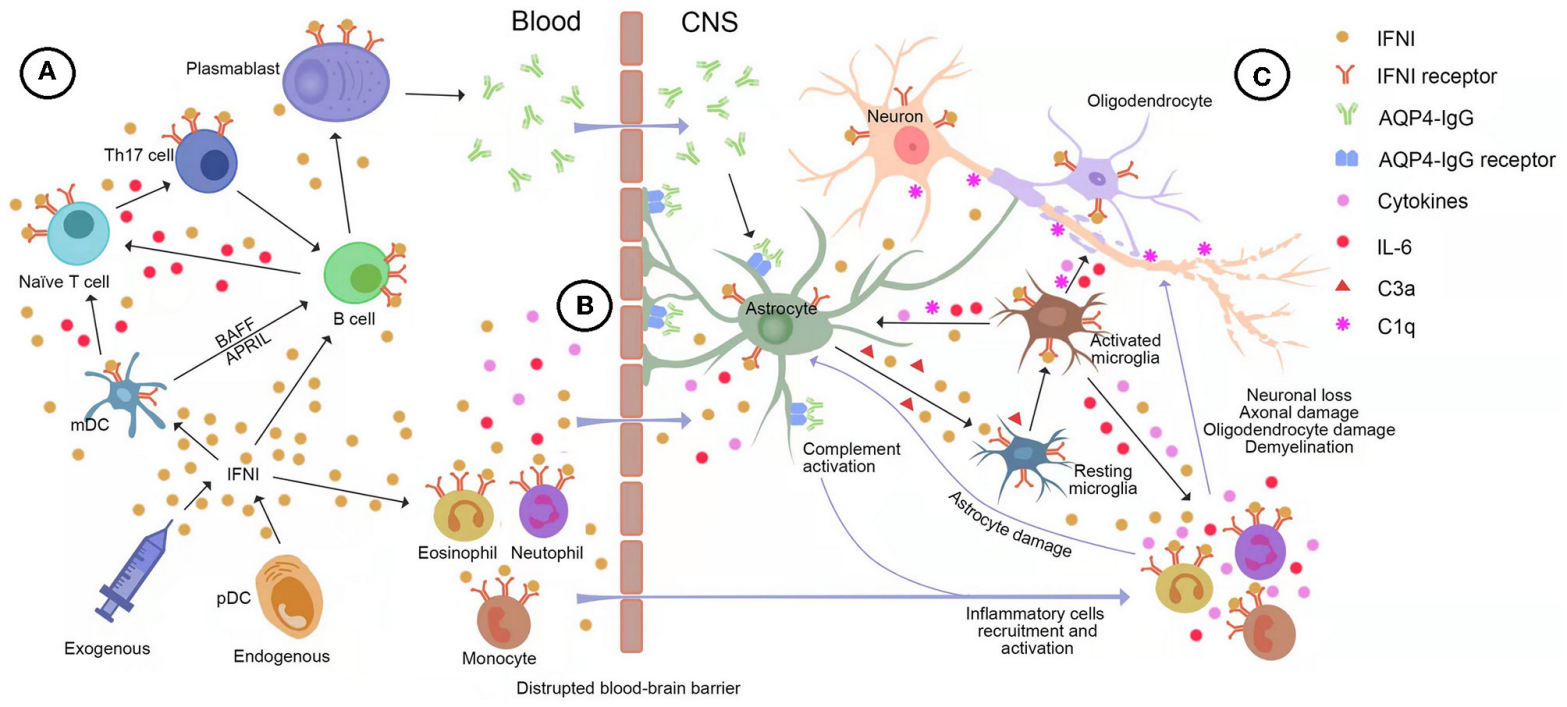
The potential role of type-I interferon (IFNI) signaling in the immunopathogenesis of neuromyelitis optica spectrum disorder (NMOSD). **(A)** Both endogenous and exogenous IFNI can drive B cells and myeloid dendritic cells (mDC) to produce large quantities of interleukin (IL)-6, which stimulates naive T cells to transform into inflammatory Th17 cells. Moreover, plasmacytoid dendritic cells (pDC) secretes IFNI into mDC, facilitating the generation of BAFF and APRIL, which are essential for the survival and maturation of B cells. In turn, inflammatory Th17 cells help B cells differentiate into AQP4-IgG-secreting plasma cells. **(B)** IFNI drives IL-6 and other pro-inflammatory molecules to disrupt and increase the permeability of the blood-brain barrier (BBB), allowing AQP4-IgG, pro-inflammatory cytokines, and immune cells to infiltrate the brain. **(C)** The IFNI-dependent astrocyte-microglia interaction drives the development of NMOSD pathology. Astrocytes are highly responsive to IFNI and the predominant source of IFNI in the central nervous system (CNS). The binding of AQP4-IgG to astrocytes induces massive production of IFNI and complement C3a, which results in the IFNI-dependent activation of microglia. Microglia respond to astroglial IFNI with the subsequent production of nitric oxide, inflammatory factors, complements, and downstream ISGs, which leads to a heightened activation state of microglia, immune cell recruitment, and complement-mediated CNS destruction. In turn, microglia secrete pro-inflammatory factors into astrocytes, especially IFNI, IL-1, IL-6, and TNF-α, leading to astrocyte activation, C1q production, and release of other pro-inflammatory factors feeding back to microglia. AQP4, aquaporin-4; APRIL, a proliferation-inducing ligand; BAFF, B-cell activating factor; BBB, brain blood barrier; C1q, Complement component 1 q; C3a, Complement component 3 a; IFN, interferon; IFNI, type-I interferon; IL-6, interleukin-6; IL-11, interleukin-11; mDC, myeloid dendritic cells; pDC, plasmacytoid dendritic cells; Th17, T helper type 17 cell; TNF-α, Tumor necrosis factor-α.

Over the last decade, research progress has contributed to the substantial expansion of our understanding of the critical role of IFNI in the immunopathogenesis of NMOSD. Both central and peripheral mechanisms of the IFNI pathway may contribute to the development of NMOSD. We provide a detailed picture of the underlying mechanism in Figure 3.

#### The Role of IFNI in Peripheral Immunity

The AQP4-IgG is generally believed to form peripherally before entering the CNS (2). It is well-established that IFNI is the most important immune mediator of peripheral immunity. Prolonged exposure to IFNI may lead to the breakdown of immune tolerance and the initiation of an autoimmune response. The IFNI, predominantly IFNα, is either endogenously produced by plasmacytoid dendritic cells (pDCs) or administered exogenously and modulates the functions of key inflammatory cells in NMOSD in the periphery. The following are the most important immune effects of IFNI (37, 38): (1) IFNI promotes the expression of MHC-I molecules, which facilitate the processing and presentation of exogenous antigens, including AQP4; (2) IFNα increases the expression of MHC-II molecules and the production of cytokines, which stimulates monocytes differentiating into myeloid dendritic cells (mDCs). Subsequently, mDCs facilitate B-Cell activating factor (BAFF) and a proliferation-inducing ligand (APRIL) generation, which are essential for the survival and maturation of B cells; (3) IFNI drives B cells and mDCs to produce large quantities of IL-6 and TGF-β, which are the key cytokines for the differentiation of Th17 cells and the suppression of Treg cell functions that lead to a protracted inflammatory response; (4) IFNI augments the differentiation of B cells into AQP4-IgG-secreting plasma cells *via* inflammatory Th17 cells and mDCs.

The IFNI also drives IL-6, CXCL10, and other pro-inflammatory molecules to disrupt and increase the permeability of the BBB, which allows AQP4-IgG, pro-inflammatory cytokines, and immune cells to infiltrate the brain.

#### The Role of IFNI in the CNS

The molecular and pathophysiological mechanisms involved in IFNI and NMOSD are being revealed *via* animal models of NMOSD. The most widely used animal models of autoinflammatory demyelinating disorders are referred to as experimental autoimmune encephalomyelitis (EAE) and can be divided into two clusters according to the passive transfer of myelin-specific TH1 or TH17 cells (TH1-EAE and TH17-EAE, respectively) (39, 40). Similar to NMOSD, TH17-EAE mice manifest ON and inflammatory demyelination in the CNS. Compared with effective treatment with IFNβ in TH1-EAE, IFNβ treatment of TH17-EAE shows significant deterioration of paralysis and increased spinal cord inflammation (39). Agasing et al. measured cytokines and inflammatory cells in TH17 mice treated with IFNβ and found that IFNI treatment of TH17-induced disease was associated with elevated serum IL-6 concentrations and TH17 cell numbers, but not with the number of neutrophils and inflammatory monocytes in the CNS. They also found that IFNIs could induce IL-6 from activated B cells to drive the pathogenic TH17 cells that play a crucial role in the occurrence and formation of NMOSD (39).

Khorooshi et al. established an animal model of NMOSD brain lesions, where typical astrocyte pathology characterized by the loss of AQP4 and GFAP was induced in mouse brains by intracerebral AQP4-IgG and human complement (huC). They observed that astrocyte pathology and associated granulocyte infiltration were reduced significantly more in IFNAR-deficient knockout (KO) mice than in wild-type mice. This result highlighted the role of IFNI signaling in the development of NMO-like pathology (41). The same team recently established a novel animal model of NMOSD-ON using intrathecal AQP4-IgG and huC. Typical astrocytopathy and NMOSD-like lesions in the optic nerve were observed in wild-type mice after intrathecal injection (42). However, NMOSD pathology was absent in IFNAR-KO mice (42). This result also strongly supported the notion that the presence of IFNI signaling is required for the development of NMOSD.

The IFNI-dependent astrocyte-microglia interaction is currently recognized as the driver of the development of NMOSD pathology (43, 44). Astrocytes are highly responsive to IFNI and the primary cells that produce IFNα in the CNS (45). The binding of AQP4-IgG to astrocytic AQP4 initiates astrocytic injury and stimulates the production and secretion of inflammatory cytokines and complement components, particularly IFNI, IL-6, and complement C3a by C3 cleavage (2). In turn, IFNI activates astrocytes to produce further inflammatory cytokines. Microglia, a critical mediator of the classical complement pathway in NMOSD pathology, can directly exacerbate neuroinflammation and promote neuroglial damage (46). Previous studies have shown that AQP4-IgG cannot directly activate microglia without astrocyte involvement. Indeed, the binding of astrocytic C3a to C3aR on resting microglia promotes microglial activation. Microglia-astrocyte crosstalk and motor impairment were shown to be absent in C3aR-deficient mice receiving AQP4-IgG, which highlights the need for the C3-C3aR axis in NMOSD pathology. Microglia respond to astroglial IFNI and C3a with subsequent production of nitric oxide, inflammatory factors, complements, and downstream ISGs, which leads to a heightened activation state of microglia, immune cell recruitment, complement cascades boost, and complement-mediated CNS destruction (43, 44). In turn, active microglia secrete abundant inflammatory factors and complements into astrocytes, which include IFNI, C1q, and IL-6, leading to a positive feedback loop. Particularly in the initial phase when there is no evident leukocyte infiltration, strong microgliosis, which is dependent on IFNIs from AQP4-IgG-binding astrocytes, corresponds to the facilitation of NMOSD-like pathology. Activated microglia significantly increases after intrastriatal injection of IFNβ in NMO mice, which subsequently exacerbates NMOSD-like pathology. In contrast, NMO-like pathology, microglia activation, and immunoreactivity markers are absent in IFNAR-KO mice that receive AQP4-IgG. In addition, the percentage of CD11c^+^ microglia was shown to be lower in IFNAR-KO mice than in control mice (46). Recent studies have found that microglia respond more strongly to IFNI than to other cell types in the CNS (33, 47). As mentioned earlier, IFNI drives the production of IL-6 and also exerts a pathogenic effect on NMOSD *via* the IL-6 pathway (39). The highly reactive microglia near the interface between the parenchyma and CSF has been identified to be closely associated with CSF IL-6 levels in patients with NMO. Correspondingly, the morphological characteristics of microglia in the IL-6 mice model are highly similar to those in patients with NMOSD (48). Therefore, it is reasonable to assume that IFNIs activate microglia *via* IL-6-driven pathology.

Taken together, IFNIs play a fundamental role in the immunopathogenesis and developmental process of NMOSD. The NMOSD is a severe neuroautoimmune disease of the CNS with high relapse and disability rates (1). Early diagnosis and rational immunotherapy strategies are crucial for improving the outcomes of patients with NMOSD. Insufficient awareness of NMOSD is a rare, yet life-threatening complication of IFNα therapy that may lead to misdiagnoses or delayed diagnoses associated with severe sequelae. Thus, screening for NMOSD should be performed as soon as possible, when patients present with early manifestations of NMOSD during IFNα treatment.

## Conclusion

Our findings highlight the potential pathogenic risk of NMOSD of IFNα treatment. We present this case report and review of the published literature to alert physicians of this rare yet devastating consequence of IFNα. Monitoring early manifestations of NMOSD during or even after IFNα treatment is vital. Moreover, prompt suspension of IFNα treatment and early initiation of guideline-directed immunotherapy strategies are essential.

## Data Availability Statement

The original contributions presented in the study are included in the article. Deidentified data, including clinical manifestations, neuroimaging data, serum tests, and cerebrospinal fluid tests, are available upon appropriate request to the corresponding author.

## Author Contributions

FF and YL contributed to the design of the study, revised the manuscript, and are responsible for the integrity and accuracy of the data. JR and NX contributed to collecting clinical data, drafting the manuscript, and reviewing the published literature. JS was the attending doctor of the patient and contributed to the acquisition and analysis of the clinical data. All authors read and approved the final manuscript to be published.

## Funding

Natural Science Foundation of Zhejiang Province of China (No. LY21H090015) and the Wenzhou Basic Scientific Research Project (No. Y20210900).

## Conflict of Interest

The authors declare that the research was conducted in the absence of any commercial or financial relationships that could be construed as a potential conflict of interest.

## Publisher's Note

All claims expressed in this article are solely those of the authors and do not necessarily represent those of their affiliated organizations, or those of the publisher, the editors and the reviewers. Any product that may be evaluated in this article, or claim that may be made by its manufacturer, is not guaranteed or endorsed by the publisher.

## Supplementary Material

Supplementary Figure 1 The timeline of the presented case.

## Abbreviations

ANA: antinuclear antibody
ANCA: anti-neutrophilcytoplasmic antibody
APRIL: a proliferation-inducing ligand
APS: area postrema syndrome
AQP4: aquaporin-4
BAFF: B-Cell activating factor
CE-MRV: contrast-enhanced magnetic resonance venography
CNS: central nervous system
CSF: cerebrospinal fluid
CT: computed tomography
CVST: cerebral venous sinus thrombosis
DID: drug-induced disease
EAE: experimental autoimmune encephalomyelitis
EDSS: Expanded disability status score
ET: essential thrombocythemia
GAD: glutamate decarboxylase
GFAP: glial fibrillary acidic protein
HCV: hepatitis C virus
HLA: human leukocyte antigen
huC: human complement
IFN: interferon
IFNI: type I interferon
IFNAR: type-I IFN receptor
IgG: immunoglobulin G
IL-6: interleukin-6
NMOSD: neuromyelitis optica spectrum disorder
MRI: magnetic resonance imaging
MS: multiple sclerosis
mDC: myeloid dendritic cells
OCT: optical coherence tomography
OCTA: optical coherence tomography angiography
ON: optic neuritis
pDCs: plasmacytoid dendritic cells
SLE: systemic lupus erythematosus.
