# Supplementary figures and images for: Case Report: Interferon-Alpha-Induced Neuromyelitis Optica Spectrum Disorder
Source: Front Neurol. 2022 Apr 25;13:872684. doi: 10.3389/fneur.2022.872684 (PMC9081932; doi:10.3389/fneur.2022.872684)

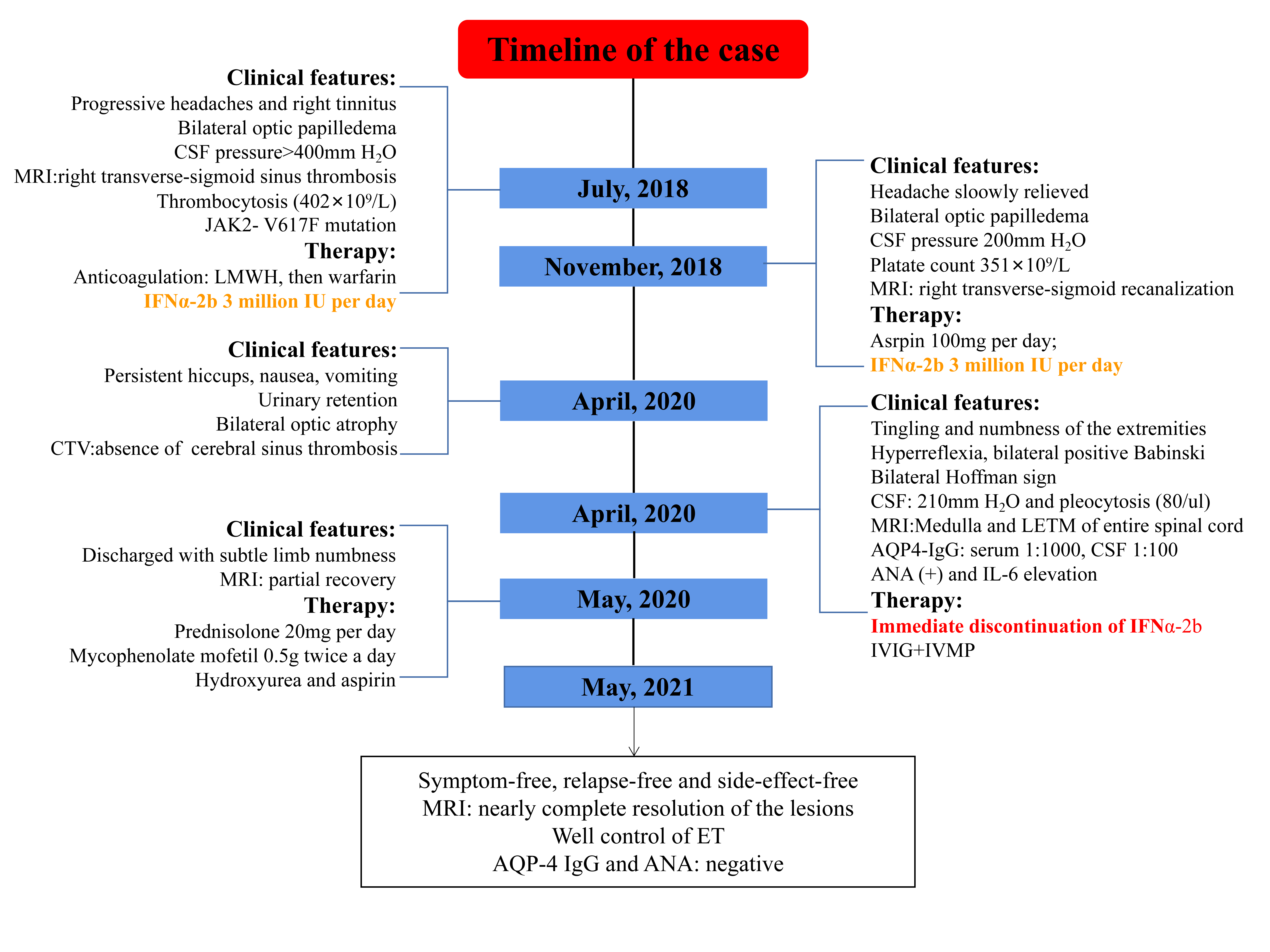

Supplement: Supplementary Figure 1 — The timeline of the presented case. [file Image_1.TIF]
